# A Comprehensive Method for the Evaluation of *Hermetia illucens* Egg Quality Parameters: Implications and Influence Factors

**DOI:** 10.3390/insects13010017

**Published:** 2021-12-23

**Authors:** Georgescu Bogdan, Dănuț Ioan Struți, Nicușor Flavius Sima, Tudor Andrei Păpuc, Boaru Anca Mihaela

**Affiliations:** 1 Department of Zoology and Ecology, Faculty of Animal Science and Biotechnologies, University of Agricultural Sciences and Veterinary Medicine Cluj-Napoca, 400372 Cluj-Napoca, Romania; georgescu.bogdan63@yahoo.com (G.B.); ptudor2008@yahoo.com (T.A.P.); 2 Department of Technological Science, Faculty of Animal Science and Biotechnologies, University of Agricultural Sciences and Veterinary Medicine Cluj-Napoca, 400372 Cluj-Napoca, Romania; nicusor.sima@usamvcluj.ro

**Keywords:** egg clutch, egg traits, counting method, black soldier fly, oviposition

## Abstract

**Simple Summary:**

Manual methods for counting insect eggs have certain shortcomings, and automatic methods often record counting errors due to confusion of objects in the visual field. There is paramount interest in research and innovation in the case of *Hermetia illucens*. Reproduction represents the vital part of the biological cycle, where the number of eggs is of significant importance. The development of a preparation method and precise egg counting, as well as an accurate technique for evaluating egg biometric parameters are the main research objectives. This precise method of egg preparation and counting proposed consists of dispersing the egg clutch under a stereo microscope and counting the eggs by the operator on a photographic capture using the Clickmaster software. The accuracy of the method is over 99.4% and the appropriate dispersion solutions were 50% glycerin and 70% ethanol. The length and width of eggs can be accurately evaluated, and positively correlated with egg weight and with the number of eggs. The methods proposed allow for a precise evaluation of the reproductive parameters of *H. illucens*, highly applicable in the selection process of adult flies as well as in the optimal breeding technology.

**Abstract:**

The significant momentum received by *Hermetia illucens* as a worldwide species is due to its biological traits and large applicability in scientific research, environmental entomoremediation, insect meal production, and for biodiesel yield. The aim of this research is to develop a method for the preparation and precise egg counting of the *H. illucens* egg clutch, as well as an accurate technique for evaluating egg biometric parameters. The precise proposed method for egg preparation and counting consists in dispersing the eggs clutch under a stereo microscope and counting the eggs on a photographic capture using the Clickmaster software. Five solution types were used to disperse the egg clutches: glycerin 50%, ethanol 70%, ethanol 80%, physiological serum 0.9% and purified water. The efficiency of the estimation method for eggs number evaluation was also tested by using the estimated egg weight as a conversion factor. The biometric parameters of single eggs (length and width) were determined using the free Toupview software. The precise method of egg preparation and counting allows for the registration of the eggs number manually identified by the operator. The appropriate dispersion solutions were glycerin 50% and ethanol 70%. The method has an error of 1.4 eggs for each 500 counted eggs, thus an accuracy of over 99.4%. The eggs number estimation method is not applicable without significant errors, the accuracy being less than 32%, due to egg heterogeneity in the clutch. Biometric parameters (length and width) are positively correlated with egg weight (r = 0.759) and with the number of eggs in the clutch (r = 0.645). In conclusion, the results clearly suggest the method of egg preparation and precise counting for an accurate evaluation of quality parameters of the *H. illucens* clutches, as well as the technique for evaluating egg biometric parameters.

## 1. Introduction

Paramount interest for the black soldier fly, *Hermetia illucens* (L.) (Diptera: Stratiomyidae) as a model organism that can easily be used for basic research is due to its advantages over other insect species, such as a shorter developmental cycle [1,2] an accelerated bioconversion of various organic substrates and its applicability in environmental entomoremediation [3,4,5]. The potential of *H. illucens* in insect farming for biodiesel yield and insect meal with high nutritive qualities for fish farming and pet food EU 2017/893 [6,7,8,9] has increasingly been explored.

The larval phase is the only stage of development when *H. illucens* consume feed, accumulating body reserves for optimal development of the organism in non-feeding stages: prepupae, pupae and adult fly. The adult fly lives for approximately 10 days in captivity using the energy reserves stored during the larval stage [10]. The capacity of ovipositing the eggs [11] is of paramount importance. The insect can reproduce only once; after the adult fly emerges, the accumulated energy reserves support the metabolic requirements of mating and egg laying [12,13,14]. Females generally lay between 300 and 1000 eggs [10,11,15], but the eggs number may be higher depending on the larval rearing medium [16,17]. Females lay eggs in sheltered, hidden spaces on a dry substrate [10,15]. The eggs are laid in clusters in the form of a package composed of overlapping layers of eggs (clutch) that adhere to each other due to a mucus that facilitates adhesion to the oviposition material [18]. As such, mating, laying eggs and larvae hatching are key moments in the artificial rearing of this insect [19,20]. The number of eggs laid is an important parameter in the selection of the insect for optimizing egg production [16,21]. In addition, higher reproductive parameters can be considered good welfare indicators, and can be used for indicating the efficiency of the larval breeding technology. Previous research has shown that the rate of growth and larval development (estimated on body weight) and the quality of eggs (weight and number) are strongly influenced by the rearing medium. Therefore, the objective aspects that should be taken into account when evaluating the efficiency of an optimal diet are the weight of eggs laid and especially the number of eggs in the clutch [16,17,22,23].

To the best of our knowledge, there is limited research analyzing the number of eggs in the clutches of *H. illucens*. Usually, this was performed by the estimation method based on the average weight of an egg used as a conversion factor [15,16,23] or by manual counting under a microscope [17,22,24] with various modifications and adjustments. Manual counting of eggs under a microscope is a time-consuming and tedious technique, especially when experiments require daily counting of hundreds of eggs [25,26,27]. The estimation method on the basis of mean weight of an egg as a conversion factor has the advantage of being able to reuse most of the eggs, but there can be experimental models in which determining the exact number of eggs is imperative.

Moreover, there are studies that analyze the efficiency of automatic and semi-automatic software for counting eggs of various insects or parasite larvae [26,28,29]. The efficiency of these methods undergoes comparison to manual egg counting. Automatic digital image recognition software can have some disadvantages, such as the inability to accurately recognize and count eggs that are clumped or overlaid, or cannot distinguish the target point from another item in the field that should not be counted. In addition, the operating interface of software can be more difficult to use. However, research findings suggest that digital image processing and egg counting software are solutions that provide good results, with acceptable errors, thus improving counting time [30]. Barbedo [31] claims that an intermediate counting variant, the so-called semi-automatic one, where items that cannot be counted by the software are subsequently counted manually, can achieve 100% accuracy in assessing the total number of eggs. The automatic counting software operates on a relatively limited number of eggs (less than 200–300 eggs) and its efficiency increases when the counting objects are well individualized and distinct. Counting errors may appear solely when the counting objects are too close to each other. *H. illucens* naturally lays eggs clumped in a compact cluster of usually 500–1000 eggs [11,15,17], so it is necessary to disperse the eggs in order for them to be clearly individualized. However, even in this case, the eggs remain close to each other. Therefore, the aim of this research was to highlight the efficiency of counting software that can be manually operated by an operator. Therefore, given this background, the research also aims at developing an optimal method for the preparation and precise egg counting of *H. illucens* clutches, as well as an accurate technique for evaluating egg biometric parameters.

## 2. Materials and Methods

### 2.1. Study Site and Biological Material Origin

The research was conducted at the University of Agricultural Science and Veterinary Medicine of Cluj-Napoca, Romania, during 2020. The biological material comes from a colony of *H. illucens* bred in an indoor laboratory for 5 years (with a mean number of 5.5 generations per year). The initial population of *Hermetia illucens* was purchased from a Greek breeding farm. Optimal medial parameters were ensured (27 ± 0.3 °C; 65 ± 4 RH; 16 h photoperiod under LED light—6000 lux) [12,24]. A population of newly emerged larvae was reared on household debris consisting of bakery waste, fruits and vegetables [2,13]. After the larval stage, the prepupae were maintained in dark containers until the imago stage, on dry leaves, ensuring the optimal temperature and humidity (27 °C ± 0.2 and 65% ± 3 RH) [11,32]. Subsequently, before the emergence of adult flies, the containers with pupae were placed in the fly rearing cages (L = 74 cm; l = 53 cm; h = 80 cm). Inside each cage, a container was introduced with an attractive substrate for oviposition, covered with a mosquito net, and the ovipositing support consisting of overlapping, 2–3 mm distanced wooden boards was placed (dimensions L = 200 mm, l = 30 mm, h = 3 mm) on top of it [33]. After oviposition, distinct clutches (<6 h-old) were collected and prepared for counting.

### 2.2. Evaluated Parameters

The study evaluates the following: (1) a precise method for counting the eggs of the *H. illucens* clutches captured in a photo, using manually operated counting software; (2) an estimation method for evaluating the number of eggs in clutches; and (3) a precise technique for evaluating egg biometric parameters (length, width).

### 2.3. Evaluation Methods of Egg Clutch Quality

#### 2.3.1. The Precise Method of Eggs Counting

The proposed method involves egg dispersion from the clutch under a stereo microscope and egg counting in a photographic capture using software that allows for the automatic recording of each egg identified by the operator. The working steps consist in harvesting the egg clutch from the support of wooden boards [33] without damaging it, followed by placing it on a glass slide to be weighed by an electronic analytical balance, accuracy 0.01 mg (model EP114C, Ohaus Corporation Pine Brook USA). The egg dispersion solution is applied over the clutch and the amount of dispersion medium used is on average 30–38 μL/clutch. The efficiency of five dispersion solutions was tested, namely: glycerin 50%—S_1_ [16], ethanol 70%—S_2_ [11], 80% ethanol—S_3_ [10], physiological serum 0.9%—S_4_, and purified water—S_5_. The glycerin 50% solution was prepared to consist of 1:1 purified water, glycerin 100% (≥99.0% purity, Merck KGaA, Darmstadt, Germany). The ethanol 70% and 80% solutions were prepared to consist of 3:7 and respectively 2:8 purified water, ethanol 100% (≥99.8% purity, Merck KGaA, Darmstadt, Germany). Physiological serum 0.9% consist of a sterile isotonic solution of sodium chloride 0.9% (Unolab Manufacturing, Madrid, Spain). Purified water 100% has a maximum 0.0005% residue (Lab-Scan Analytical Sciences, Gliwice, Poland).

As such, a number of 10 clutches were analyzed for each of the five-dispersion media (*n* = 10/solution). The clutches were clearly individualized and distinct, offering the certainty that each originated from a single female. Variable volumes of solution (depending on clutch size, usually 10 microliters of medium/10 mg of eggs) were placed over and around the clutches using a micropipette. A microscope slide cover glass was placed over the eggs and was lightly pressed by a glass rod with a rubber tip. Thus, the eggs were dispersed out of the clutch, obtaining a single evenly distributed egg layer, without overlaps or agglomerations.

The eggs dispersed on the slide were analyzed with a binocular stereo microscope (Alpha model, 10×–40× magnification, Novel Industries, Ambala, India) using different levels of magnification. The eggs were photographed by a digital camera (20 megapixels resolution) attached to the binoculars. The images were downloaded to a PC and the eggs were counted using the ClickMaster2000 software, which is free to access and download and use (https://www.thregr.org/~wavexx/software/clickmaster2000).

**Figure 1:**
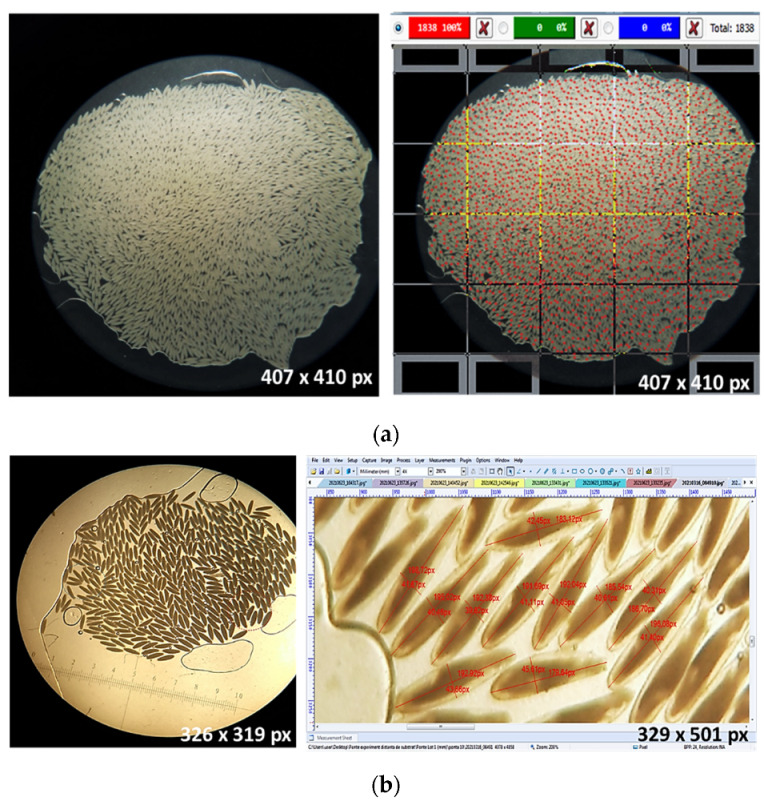
Evaluating the egg clutch of *Hermetia illucens* (<6-old): (**a**) dispersion eggs from the clutch and counting byClickMaster software; (**b**) evaluation of the egg length and width by Toupview software (red bars = length and width of egg).

The software interface allows for the identification and registration of each egg marked by a mouse click on the image, thus counting all the eggs. In this way, each egg can be quantified with maximum precision, finally obtaining the total number of eggs from each clutch (Figure 1).

The optimal egg dispersion medium was assessed in terms of its efficiency to ensure uniformity, compactness and a clear dispersion of the eggs, as well as in terms of the precision and operability of the counting process. Egg preparation and counting time was recorded. Furthermore, the precision and accuracy of the counting method was evaluated by simultaneous counting performed by three operators on each of the 10 clutches/dispersion medium.

#### 2.3.2. The Estimation Method for Egg Counting

The efficiency of an estimation method for the assessment of eggs number by the use of the mean weight of an egg as a conversion factor was determined. The estimated average egg weight was determined by weighing a group of 10, 20, 30, 40, and 50 eggs from the whole clutch (with an electronic balance, 0.01 mg accuracy). The estimated mean egg weight of each group was used as a conversion factor to estimate the number of eggs in the clutch. Therefore, the weight of the whole clutch was related to the estimated weight of the egg, thus resulting in the estimated number of eggs in the clutch. The eggs number obtained by the estimation method was compared with the real number of eggs obtained through the precise egg counting method (described above). The estimated egg weight of each of the previous egg groups (10 … 50) was compared with the average egg weight obtained by the precise counting method).

### 2.4. Assessment of Egg Biometry

The technique for evaluating egg biometric parameters consisted in the determination of the length and width of 50 eggs, randomly selected from each analyzed clutch (*n* = 50 clutches ranmdomlyselected from the colony). The measurements were performed using the Toupview ^®^ software (https://levenhuk-toupview.software.informer.com/3.7). Egg preparation for this method involved the same steps described above for the precise method of counting eggs. A standard scale of 0.1 mm was placed near the eggs, as a reference unit for the dimension conversion from pixels to millimeters. After capturing a photo image with a stereo microscope (Alpha model, 10×–40× magnification), it was uploaded on a PC in the Toupview software. From the “Measurements” section, a line was drawn in the widest side of the egg (considered the maximum egg width) and in the longest side (considered the maximum egg length) (Figure 1). The resulting values were automatically recorded in the database of the software.

All 50 egg clutches used for the analyses of egg biometric parameters were subjected to counting by the precise method of counting, using 70% ethanol as a dispersion medium. The clutches were categorized as following: clutch with eggs number less than 500 (A); clutch with eggs number ranging between 501 and 1000 (B); clutch with eggs number ranging between 1001 and 1500 (C); clutch with eggs number over 1500 eggs (D). The evaluated parameters were: whole egg weight (mg), total number of eggs, average egg weight (mg), egg length (mm), egg width (mm). Egg dimensions were correlated with the average egg weight and whole clutch weight, respectively with the number of eggs in a clutch. Fifty eggs from two distinct regions in opposite areas of the clutch were randomly identified and evaluated for length and width, to detect differences between eggs in different areas of the same clutch.

### 2.5. Statistics

The differences between the quality parameters of clutches (weight, total number of eggs) and of individual eggs (egg weight, egg length, egg width) evaluated between the different clutch size categories were subjected to an ANOVA single-way test at a significance level of 5%, followed by the Tukey HSD as a post-hoc test (*p* < 0.05). The Pearson correlation was used to establish the correlation coefficients between the clutch quality parameters (total weight and number of eggs, single egg weight). The accuracy of the egg counting method by ClickMaster, tested by three different operators, was analyzed by the Student’s test (*t*-test) (*p* < 0.05). The results of the efficiency between the precise counting method and the estimation method are expressed as a percentage, because it was not possible to apply a statistical test. All data are presented as mean ± standard error (se).

## 3. Results and Discussion

### 3.1. Method of Precise Egg Counting

First and foremost, the evaluation of the eggs number by using this method allows for the precise quantification of each egg, if the dispersed eggs are distributed uniformly and do not overlap. Clutch eggs, where overlapping layers are encountered, need to be dispersed in a uniform layer where each egg is well highlighted and the counting is achieved with maximum accuracy. Barros et al. [18] reported that eggs of *H. illucens* were covered with a mucus that facilitated their arrangement in a package, adhering to the laying substrate. From this perspective, the action capacity of the dispersion medium is essential, in terms of mucus dissolution. Regardless of the dispersion medium used, the work time required was calculated for each step: from the collection of the eggs from the insect cage and placing them on the lamella (60–90 s), application of the dispersion medium and the slide cover (40–60 s), and focusing and image capture (60–90 s).

**Figure 2:**
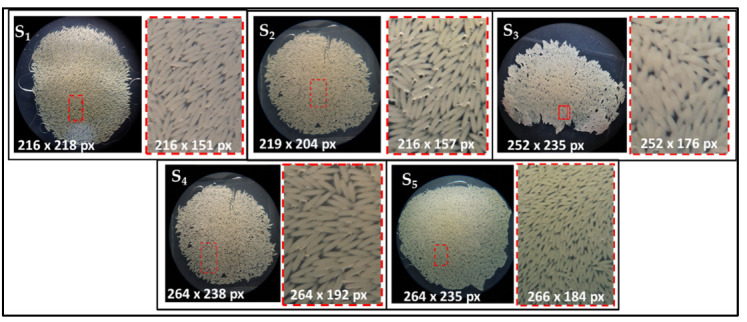
Quality of *Hermetia illucens* egg dispersion using different media: S_1_—glycerin 50%; S_2_—ethanol 70%; S_3_—ethanol 80%; S_4_—physiological serum 0.9%; S_5_—purified water.

For instance, glycerin 50% (S_1_) ensured a uniform, single-layer distribution of the eggs. Distinct individualization and easy counting are possible only in the case of fresh eggs (Figure 2) (less than 6 h after deposition) and becomes more difficult afterwards. Subsequently, the compact distribution of eggs takes place when the embryonic development processes are more advanced (18–36 h after deposition), which makes it difficult to distinguish eggs, thus generating frequent counting errors. Sometimes, there may be moderate numbers of air bubbles, which impede the individualization of the eggs older than 24 h.

Considering ethanol 70% (S_2_), it allows for an optimal and clear egg distribution. Eggs can be counted without errors, regardless of the stage of their embryonic development. Air bubbles may be present, but are small in number and size and do not influence the accuracy of the count (Figure 2).

Additionally, ethanol 80% (S_3_) leads to satisfactory conditions for egg counting, as well. However, due to the volatile properties of the high concentration, the optimal dissociation of eggs does not occur. More often, eggs are compact, forming small agglomerated masses. Air bubbles are rarer than in 70% ethanol and, in some cases, the dispersion medium is clear and completely free of bubbles (Figure 2).

Physiological serum 0.9% (S_4_) allows for an efficient dispersion of eggs from the clutch and disposes the eggs in a single layer. A good disposition for egg counting can be achieved with fresh eggs, which can be easily identified. In older clutches, due to the agglomeration of eggs in a compact package, it is difficult to identify each egg. At the same time, numerous air bubbles of a relatively large size frequently appear and can affect counting accuracy. These bubbles are usually distributed between the eggs and disturb the counting process, especially in the case of older eggs (Figure 2).

Similarly, purified water (S_5_) allows for the egg clutch to be distributed in a single layer, but the dispersion is reduced. The eggs remain in close contact, creating a relatively compact mass, which leads to counting errors for eggs with a more advanced embryonic development. Numerous air bubbles of varying sizes appear in most samples, the majority being large. The distribution of air bubbles in the gaps between eggs allows older eggs to dissociate from their neighboring eggs (Figure 2).

Egg dispersal is considered a very important step for obtaining a distinct individualization of each egg, thus directly influencing the accuracy in obtaining the real number of eggs in a clutch. In this regard, Friedland et al. [34] developed an imaging-based technique that counts and measures oocytes, showing that the most important factor in reducing the error rate (less than 1%) and improving counting accuracy was evenly dispersing the eggs before taking the image of the sample. In our method, egg dispersing is performed mechanically by slightly pressing the slide cover over the clutch, which can generate the breaking of several numbers of eggs, if the maneuver is performed carelessly and by applying too much force. The spillage of egg contents alters the transparency of the dispersion medium and can generate counting errors. In addition, the physical properties of the dispersion media used directly influence the spacing of the eggs and allow for their precise individualization. For fresh eggs, it allows for an easy identification, but in more advanced stages of embryonic development, individualization is frequently compromised, counting errors being inherent.

Literature studies report ethanol 70% and 80% to be a medium utilized for the separation of eggs from the clutch pack, and for the individual egg counting of *H. illucens*. In this regard, Nakamura et al. [11] soaked clutches of different sizes in 70% ethanol solution for egg separation. Tomberlin et al. [10] introduced clutches in vials with 80% ethanol to be counted later, while Bertinetti et al. [16] used a medium of glycerin and water 1:1 to disperse and facilitate egg counting under a binocular microscope. In applying our method of egg dispersion, the solution was added with a micropipette over the whole clutch, and the ethanol (S_2_ and S_3_) and glycerin (S_1_) diffused spontaneously in the egg mass, while the purified water (S_5_) and physiological serum (S_4_) remained above the egg mass and diffused after applying the glass slide. It was also found that ethanol 80% spreads the eggs excessively and does not disperse efficiently, often leaving eggs agglomerated in smaller packets or displacing them in large numbers from the microscope field, making it impossible to capture an image with all the eggs. Physiological serum 0.9% maintains the cohesion of the egg mass. Therefore, in some situations, counting is difficult and errors can occur. The most effective solutions for dispersing the egg mass proved to be S_2_ (70% ethanol) and S_1_ (50% glycerin).

Egg counting using the ClickMaster software was facile, undemanding and easy to perform because each egg was marked with one click, individualized with a color point and registered by the program (Figure 1). Depending on the number of eggs in the clutch, the longer time before deposition and the dispersion medium used, the counting time of the eggs takes from 5 to 7 min for every 500 eggs counted. By using the S_1_ and S_2_ dispersion media, a counting error of 1.4 eggs for every 500 counted eggs occurred. Nakamura et al. [11] photographed the egg clutch of *H. illucens* under the microscope and counted them individually in the printed picture. Jucker et al. [22] counted eggs of *H. illucens* by using a stereomicroscope while, recently, Macavei et al. [24] reported that eggs of *H. illucens* were counted manually under a microscope using a teasing needle. Even though the manual counting of eggs is still used in many cases, it is time-consuming, it can present difficulties in individualizing each egg, and has a higher rate of human error; moreover, some eggs can be destroyed when using instruments to separate them from each other (G. Bogdan, unpublished data).

The use of automatic programs for counting insect eggs or parasite larvae shows that, in most cases, the differences between the values resulting from the software count were insignificantly different from those obtained by manual counting. For instance, Gaburro et al. [26] report the “iCount” software for automatic counting of *Aedes aegypti* eggs as a method three times faster than manual counting, with insignificant counting errors (*p* > 0.05) that occur in the case of close or joined eggs. A method proposed by Mello et al. [25] involves the automatic counting of *A. aegypti* eggs by image processing, where the results showed errors of 7–8%, which were considered satisfactory by the authors. For the same species, Garcia et al. [27] counted the eggs by digital methods using a camera coupled with a magnifying glass for acquiring images. The authors presented a deficiency of the method in the case of 10% of the tested images due to the high density of the eggs or the presence of black elements that resemble software-counted mosquito eggs. Additionally, Silva et al. [35] used a method of optical scanning and automatic counting of mosquito eggs, showing that it had an error of 2.67% compared to the manual method, but also a halved working time. The system was based on an optical scanning platform, a man-machine interface, and software for mosquito egg counting. Also, Mollahosseini et al. [28] created automatic software for counting *Anopheles* egg batches. The results obtained by automatic counting were close to the values obtained following manual counting, while Waithe et al. [29] analyzed the efficiency of the QuantiFly software for the automatic counting of *Drosophila melanogaster* (Diptera: Drosophilidae) eggs compared to manual counting in digital images, obtaining an efficiency of only 85–94%, depending on the egg deposition medium. The potential of smartphones, as relatively sophisticated, inexpensive and portable devices, to perform image analysis for parasite faecal egg counting was studied by Slusarewicz et al. [36]. Subsequently, research was conducted to demonstrate the accuracy and precision of two smartphone prototype software for automated parasite egg counting compared with the manual method, yielding no significant errors (*p* > 0.05) [37].

### 3.2. Estimation Method of Eggs Number

The evaluation by estimation of the eggs number from the 50 clutches revealed a value similar (*p* > 0.05) to that obtained by precise counting only in five cases (representing 10% of the total), when the estimated weight of an egg was based on the mean of 10 eggs. There were no cases where the real number of eggs in a clutch (obtained by precise counting) corresponded without significant error (*p* < 0.05) to the estimated number of eggs, when the estimated weight of an egg was obtained as a mean of 20 weighed eggs. A number of 3 clutches (6% of total) had an estimated eggs number value similar to the real value (*p* > 0.05), when the estimated egg weight used as conversion factor resulted from weighing 30 eggs, and only one correct estimate (2% of total) when the mean was performed on 40 eggs. Most cases where the estimated number of eggs was similar (*p* > 0.05) to the real number of eggs (8 clutches—16% of total) occurred when the estimated weight of the egg as a conversion factor resulted from weighing 50 eggs. Therefore, in the case of 16 clutches (32% of total) the estimated number of laid eggs was similar to the real one (*p* > 0.05).

In the case of 14 egg clutches (28% of total), the progressive egg weight assessments from 10 eggs to 50 eggs followed an expected direction of gradual increase in the degree of accuracy. Of these, only 6 clutches (12%) finally had an estimated number that did not significantly differ from the real number (*p* > 0.05). The mean egg weight was the same in all groups of weighed eggs (from 10 to 50 eggs) in only four cases (8%). It is to be concluded that eggs number determination in *H. illucens* clutches through the estimation method is not applicable without significant errors. The values of the estimated number of eggs did not correspond to the real values obtained by the precise counting method for 68% of clutches. Twelve percent of the egg clutches presented deviations exceeding by more than 100% the real eggs number value. Seldom, an approximate number of eggs is sufficient, while there are studies where the total number of eggs was estimated based on the weight of a reduced number of eggs [15,16,23]. However, when the number needs to be exact, it is recommended to use the precision method described.

The estimated weight of an egg based on a group of 10 and 20 weighed eggs showed that a value similar (*p* > 0.05) to the real average weight of the egg, resulted by dividing the weight of the whole clutch by the real number of eggs (determined by precise method), was obtained in only 5 cases (10% of total). The estimated weight of the egg resulting from the weighing of 30 eggs led to the obtaining of correct results (*p* > 0.05) in only 7 cases (14%). When determining the estimated weight of the egg based on 40 eggs, the results obtained were similar (*p* > 0.05) to those from the evaluation of 10 and 20 eggs (10%). The most correct evaluations (10 egg clutches—20%) were obtained when the estimated weight of the egg resulted from the weighing of 50 eggs. There were solely two cases (4% of total) where the estimated egg weight was similar during the 5 progressive determinations (from 10 to 50 eggs) and at the same time, similar to the real egg weight (*p* > 0.05).

### 3.3. Physical Quality Parameters of Hermetia illucens Eggs

#### 3.3.1. Biometric Egg Parameters

The increased number of eggs in the clutch is correlated with the increase in weight, the differences between the different categories of clutches being statistically supported (Table 1). Our previous research demonstrated that these aspects depend on different experimental factors and/or treatments applied in larval feeding [14,17].

Egg length and width increase gradually and constantly from one egg clutch category to another. The egg clutches with a total number of up to 500 eggs have eggs with a mean length of 0.8839 and a width of 0.2176 as the lowest values, and corresponding to eggs with the lowest weight (0.0227 mg/egg) (Table 1). These values increase linearly in the case of clutches with a number of over 1500 eggs, where the length of the egg was 0.9843 mm and the width 0.2381 mm (Table 1).

The differences in egg length are significant (*p* < 0.05) between each category of egg clutches (Table 1). Our findings are consistent with those obtained by Barros et al. [18], who reported a length of *H. illucens* eggs between 1 and 1.4 mm and the diameter of the eggs between 0.4 and 0.6 mm. For instance, a number of 33 eggs was found from different clutches that had a length of over 1 mm, which is within the limits presented by the authors, but not for egg width range, where the highest value found was 0.275 mm.

The width of the eggs analyzed in the category C recorded an average value of 0.2165 mm, being smaller (*p* > 0.05) than in the case of both previous categories. However, egg width in the first three clutch categories (A–C) (<500, 501–1000 and 1001–1500) was smaller (*p* < 0.05) than that of the eggs belonging to the D clutch category (Table 1).

The progressive tendency to increase egg weight is also confirmed by the evolution of biometric parameters, namely egg average length and width. The clutches with a number of up to 500 eggs exhibited the lowest egg weight (0.0227 mg/egg), which underwent a slight increase (*p* > 0.05) to 0.0234 mg/egg in the category of 500–1000 egg-clutches. As for the maximum egg weight, it stabilized around 0.0275 mg/egg in the category of with 1000–1500 egg-clutches, a value close to the average egg weight in the 1500+ category, i.e., 0.270 mg/egg (Table 1). The Pearson correlation coefficients showed that the average egg weight growth trend significantly correlated with the egg weight (r = 0.759) and with the number of eggs in the whole clutch (r = 0.645).

According to obtained data, it can be estimated that clutches weighing up to 10.0 mg have a number of less than 500 eggs; while a clutch weighing between 10–20.0 mg has between 500–1000 eggs; a clutch weighing 30–40.0 mg has 1000–1500 eggs and those weighing more than 40.0 mg generally have more than 1500 eggs.

#### 3.3.2. Factors Influencing Accuracy in Evaluating Egg Biometric Parameters

An accurate assessment of egg biometric parameters is possible if clutches with a small eggs number, i.e., up to 500 eggs, which are dispersed in 70% ethanol are assessed. For clutches with a larger eggs number (over 1000 eggs), the compact distribution of eggs impedes size assessment. In general, the accuracy in evaluating egg length does not present any issues because the small size of the distal extremities is clear, the precise delimitation of the two points being relatively easy to identify (Figure 1). When measuring eggs width, difficulties in an accurate and correct evaluation often occur. The large surface area of the flanks causes frequent contact with neighboring eggs, making it difficult to correctly identify the boundary point. This phenomenon is common in the case of clutches with a large number of eggs, or when the dispersion medium has not effectively delimited the eggs.

**Figure 3:**
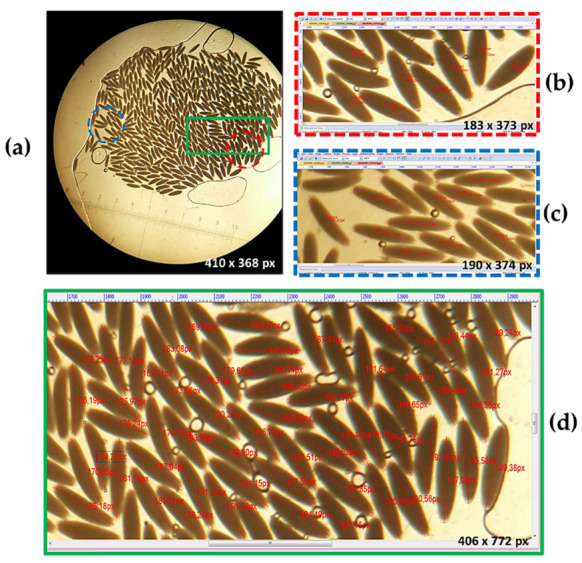
Variable egg sizes in different areas of the same clutch (**a**) egg with mean length of 0.889 mm and width of 0.232 mm (**b**); egg with mean length of 0.796 mm and width of 0.191 mm (**c**); egg with values of length and width intermediate between the two areas (**d**).

A more precise evaluation of the biometric parameters was possible in the eggs from the periphery in most of the analyzed egg clutches. They are usually fairly far apart from neighboring eggs, to allow accurate measurement. It is also essential to randomly measure several eggs from different areas of the clutch, in order to obtain relevant average values. The analyzed eggs showed a great heterogeneity in terms of egg size. Thus, eggs with different length and width (*p* < 0.05) were present in the same clutch (Figure 3). The dissociation of eggs into two groups of sizes in the same clutch showed the presence of differences (*p* < 0.05) both in the case of egg length (0.889 vs. 0.796 mm) and of width (0.232 vs. 0.191 mm). Numerous eggs with intermediate values were observed between the two analyzed areas (Figure 3).

The variability of the eggs size inside the clutch, which correlates with egg weight, explains the contradictory data obtained in the case of the estimation method for the number of eggs in the clutch. The large-scale variability in egg weight in the same clutch also explains the intrapopulation variability found in previous experiments on the adult flies that benefited from identical feeding and maintenance conditions throughout their life cycle [17].

Data accuracy in the process of evaluating the general clutch features is influenced by the stage of embryo development. Freshly laid eggs, from the first 6 to 12 h, are opaque, white, well displayed and allow for both easy counting and easy measurement. Physically, the eggs from the fresh clutch of *H. illucens* are characterized by an elongated shape, similar to a rice grain, with rounded and slightly elongated heads and with a milky white, cream color. Similar to our findings, Barros et al. [18] report that the color of fresh eggs varies from cream-white in the first hours to yellowish as the embryo develops.

After approximately 18 h, the embryo development processes lead to the gradual narrowing of the opaque area and the highlighting of clear semi-transparent areas, which make the individualization difficult for compactly arranged eggs. As such, eggs that have exceeded 24 h of age have a cloudy and agglutinated appearance of the internal medium and, at this stage, the eggs cannot be counted without errors, biometric measurements being also difficult to perform in many cases. A clutch with compact eggs, which have exceeded 48 h of life, cannot be counted and biometric measurements are impossible.

During the exhaustive evaluations of some freshly laid eggs, particular situations were identified, when eggs were transparent and in obvious contrast with neighboring eggs. A possible explanation may be the lack of vitellus, which suggests an altered process of ovogenesis, or disorders that are asserted in the vitellogenic stage [38,39].

Additionally, in some egg clutches, the presence of a variable number of eggs retaining the similar characteristics of fresh eggs and distributed among viable eggs was observed. To our knowledge, these eggs are unfertilized, which is why embryo development has not been initiated. Several egg clutches where some eggs had obvious gaps in the stage of embryo development were identified. Most eggs have specific characteristics of freshly laid (<6 h) eggs. However, there is a relatively small number of fresh eggs where the process of embryonic development was in an obviously more advanced stage and differences were contrasting.

Some eggs halted in their evolution were observed in egg clutches with an advanced development stage (24–48 h from the deposition). Most of these eggs underwent normal development, but eggs where the internal medium seemed to have entered an advanced liquefaction process were also distinguished and can be relatively easily dissociated from the rest. For instance, eggs older than 48 h, in the advanced stage of embryo development change their color, acquiring a straw-yellow to pronounced yellow and yellow-orange appearance.

Our proposed method of preparation and precise egg counting is a semi-automatic technique that allows for a quick and accurate egg counting compared to other methods, as Clickmaster software is a useful, free tool, which has an advantageous and easy to use interface. All stages of the proposed method, namely preparation and eggs counting with the Clickmaster software, were validated as a semi-automatic method of *H. illucens* egg counting using image processing. Moreover, the biometric parameters of the eggs can be easily determined by using the free and easy to use Toupview software. These methods allow for the determination of both the accurate number of *H. illucens* eggs and of biometric egg parameters, aspects that bring a significant contribution to improving the knowledge of the biological particularities of the species. Thereby, it is considered that by the precise counting of the eggs number, some aspects related to the precise evaluation of the reproductive parameters of *H. illucens* are solved, to be applied in the selection process of adult flies and in industrial rearing technology. For the management of egg densities per gram of diet that can be handled with almost 100% precision, it is necessary to mix several clutches followed by the individual counting of the eggs using the precise method, because any estimation induces errors due to the heterogeneity of the eggs in a clutch and between clutches.

The determinations of the weight and the number of eggs in the clutch of *Hermetia illucens* aimed to evaluate some limiting growth factors, considering reproductive parameters as a sensitive indicator for the effect of different feed sources in larvae [22,23] and adult flies [11,16,24], temperature and humidity [10] and light for adults [40]. Using this method in a mass rearing protocol can have advantages in improving the evaluation of reproductive performances as well as in the selection process of flies, or to create new opportunities. To our knowledge, in this field, the method represents a novelty. Therefore, additional new studies are required henceforth. Being an improvement, the method can be applied in laboratory research using this species, where limiting factors of species growth can be quantified through the effects on the reproductive performance of *Hermetia illucens* adults. In addition, the precise method applicability improves the experimental conditions concerning the reproductive parameters of the species, because it allows the processing of a large number of freshly laid eggs in a short time (several hours), and after the preparation and image capture of the eggs, they can be counted later, over several days.

## 4. Conclusions

In conclusion, the method developed for the accurate and precise assessment of the number of clutch eggs can be applied, even with a counting accuracy of 100%. The most efficient dispersion media proved to be 70% ethanol and 50% glycerin, which ensure easy egg counting and measurement. The maximum precision evaluation of the eggs, both from a numerical point of view and from the perspective of biometric measurements, can be performed only in the case of fresh eggs, namely in the first 10–12 h after oviposition. Accurate measurement of egg size is possible especially in the peripheral areas of egg clutches with a large number of eggs, and in any area of small egg clutches, with a number of up to 500 eggs. Egg length and width are correlated with their weight, and their values increase progressively with the increase of the eggs number in the clutch. The estimation method of clutch eggs number by assessing the average weight of a variable number of eggs and relating it to the total weight of the clutch is applicable to *H. illucens* only with errors in most cases. The methods could be practically applied in the optimizing of the industrial rearing of larvae and the practice of the selection of adult flies, as well as for the objective evaluation of different life cycle influence factors, and for the assessment of biologically-active environmental pollutants. In addition, a potential applicability of the precise evaluation of egg quality parameters could consist in the evaluation of the biological risk generated by the exposure to different environmental contaminants.

## Figures and Tables

**Table 1:** Quality parameters of different sizes of *Hermetia illucens* egg clutch (mean ± se).

Specification	Categories of Egg Clutch				Anova Single-Way	
	A	B	C	D	*F*	*p*-Value
Clutch weight (mg)	8.65 ± 0.52 ^a^	17.37 ± 0.76 ^b^	35.07 ± 1.79 ^c^	44.7 ± 1.20 ^d^	124.97	0.000
Eggs number in clutch	378.80 ± 14.10 ^a^	740.53 ± 24.23 ^b^	1272 ± 41.75 ^c^	1659 ± 93.61 ^d^	166.40	0.000
Egg weight (mg)	0.0227 ± 0.001 ^a^	0.0234 ± 0.001 ^a^	0.0275 ± 0.001 ^b^	0.0270 ± 0.001 ^b^	8.333	0.000
Egg length (mm)	0.8839 ± 0.005 ^a^	0.9207 ± 0.004 ^b^	0.9748± 0.005 ^c^	0.9843± 0.004 ^dc^	56.44	0.000
Egg width (mm)	0.2176 ± 0.002 ^a^	0.2228 ± 0.001 ^a^	0.2165 ± 0.001 ^a^	0.2381 ± 0.003 ^b^	14.53	0.000
*n* (clutch)	11	23	10	6	-	-

se = standard error; F = calculated value; ^a–d^ = different superscripts in the same row show significant differences (*p* < 0.05); A = category of clutch with eggs number less than 500; B = category of clutch with eggs number ranging between 501 and 1000; C = category of clutch with eggs number ranging between 1001 and 1500; D = category of clutch with eggs number over the 1500 eggs; *n* = number of analyzed clutches.

## Data Availability

The data supporting the reported results are in the possession of the authors.
